# Supplementary material for: Predation and fragmentation portrayed in the statistical structure of prey time series
Source: BMC Ecol. 2009 May 6;9:10. doi: 10.1186/1472-6785-9-10 (PMC2689204; doi:10.1186/1472-6785-9-10)
Supplement: Additional file 2 — Voles and related classes ODDox Documentation. ODDox documentation of the agent-based model (ALMaSS) applied by Hendrichsen et al. The documentation is started by activating main.html. [file 1472-6785-9-10-S2.zip › Vole_ODDox/tole__declaration_8h.html]

ALMaSS ODDox: tole\_declaration.h File Reference

- Main Page
- Related Pages
- Classes
- Files

# tole\_declaration.h File Reference

|  |  |
| --- | --- |
|  |  |
| Enumerations | |
| enum | TTypesOfLandscapeElement {     tole\_Hedges = 0, tole\_RoadsideVerge, tole\_Railway, tole\_FieldBoundary,     tole\_Marsh, tole\_Scrub, tole\_Field, tole\_PermPastureLowGrazing,     tole\_PermanentSetaside, tole\_PermPasture, tole\_NaturalGrass, tole\_RiversidePlants,     tole\_PitDisused, tole\_RiversideTrees, tole\_DeciduousForest, tole\_MixedForest,     tole\_ConiferousForest, tole\_YoungForest, tole\_StoneWall, tole\_Garden,     tole\_Track, tole\_SmallRoad, tole\_LargeRoad, tole\_Building,     tole\_Urban, tole\_ActivePit, tole\_Freshwater, tole\_River,     tole\_Saltwater, tole\_Coast, tole\_HedgeBank, tole\_BeetleBank,     tole\_Heath, tole\_Orchard, tole\_UnsprayedFieldMargin, tole\_Foobar   } |

---

## Enumeration Type Documentation

enum TTypesOfLandscapeElement

**Enumerator:**
:   |  |  |
    | --- | --- |
    | *tole\_Hedges* |  |
    | *tole\_RoadsideVerge* |  |
    | *tole\_Railway* |  |
    | *tole\_FieldBoundary* |  |
    | *tole\_Marsh* |  |
    | *tole\_Scrub* |  |
    | *tole\_Field* |  |
    | *tole\_PermPastureLowGrazing* |  |
    | *tole\_PermanentSetaside* |  |
    | *tole\_PermPasture* |  |
    | *tole\_NaturalGrass* |  |
    | *tole\_RiversidePlants* |  |
    | *tole\_PitDisused* |  |
    | *tole\_RiversideTrees* |  |
    | *tole\_DeciduousForest* |  |
    | *tole\_MixedForest* |  |
    | *tole\_ConiferousForest* |  |
    | *tole\_YoungForest* |  |
    | *tole\_StoneWall* |  |
    | *tole\_Garden* |  |
    | *tole\_Track* |  |
    | *tole\_SmallRoad* |  |
    | *tole\_LargeRoad* |  |
    | *tole\_Building* |  |
    | *tole\_Urban* |  |
    | *tole\_ActivePit* |  |
    | *tole\_Freshwater* |  |
    | *tole\_River* |  |
    | *tole\_Saltwater* |  |
    | *tole\_Coast* |  |
    | *tole\_HedgeBank* |  |
    | *tole\_BeetleBank* |  |
    | *tole\_Heath* |  |
    | *tole\_Orchard* |  |
    | *tole\_UnsprayedFieldMargin* |  |
    | *tole\_Foobar* |  |

```
00013              {
00014               tole_Hedges=0, // 130
00015               tole_RoadsideVerge, // 13
00016               tole_Railway, // 118
00017               tole_FieldBoundary, // 160
00018               tole_Marsh, // 95
00019               tole_Scrub, // 70
00020               tole_Field,  // 20 & 30
00021               tole_PermPastureLowGrazing, // 26
00022               tole_PermanentSetaside, // 33
00023               tole_PermPasture, // 35
00024               tole_NaturalGrass, // 110   //tole 10
00025               tole_RiversidePlants, // 98
00026               tole_PitDisused, // 75
00027               tole_RiversideTrees, // 97
00028               tole_DeciduousForest, // 40
00029               tole_MixedForest,     // 60
00030               tole_ConiferousForest, // 50
00031               tole_YoungForest, //55
00032               tole_StoneWall, // 15
00033               tole_Garden, //11            // tole 20
00034               tole_Track,  // 123
00035               tole_SmallRoad,  // 122
00036               tole_LargeRoad,  // 121
00037               tole_Building, // 5
00038               tole_Urban, // 10
00039               tole_ActivePit, // 115
00040               tole_Freshwater, // 90
00041               tole_River, // 96
00042               tole_Saltwater,  // 80
00043               tole_Coast, // 100          // tole 30
00044                           tole_HedgeBank, // 140
00045                           tole_BeetleBank, // 141
00046               tole_Heath, // 94
00047               tole_Orchard, //56
00048               tole_UnsprayedFieldMargin, // 31
00049               tole_Foobar // 999   !! type unknown - should not happen
00050               // Don't use this one when communicating with the landscape
00051               // model, it will trip an error in most cases!
00052               // --FN-- 24/11-2000.
00053              }
```

---

Generated on Thu Jan 22 14:13:45 2009 for ALMaSS ODDox by 
 1.5.6
